# Consumption of aphrodisiac drugs without prescription among men in Saudi Arabia: cross-sectional study

**DOI:** 10.1016/j.jsps.2024.101955

**Published:** 2024-01-10

**Authors:** Sameer Hamdy Hafez, Sadeq Abdo Mohammed Alwesabi, Elwaleed Idris Sagiron, Hanan Saad Abdullah Alwadei, Abdalla MohamedAhmed Osman Abdalla, Elsadig Eltaher Hamed ِAbdulrahman, Nahid Khalil Elfaki, Noha Ahmed Mohamed, Mohammed Abdulrahman Alshahrani, Ahmad A. Alshehri, Mohammed Jamaan Alzahrani, Amna Mohammed Idris, Mohamed Gamal Elsehrawey, Mohammad El-Nablaway, Ateya Megahed Ibrahim

**Affiliations:** a Community Health Nursing, Beni-Suef University, Faculty of Nursing, Egypt; b Community and Mental Health Nursing, College of Nursing, Najran University, Najran, Saudi Arabia; c Department of Medical and Surgical Nursing, Faculty of Nursing, Najran University, Najran, Saudi Arabia; d Department of Clinical Laboratory Sciences, Faculty of Applied Medical Sciences, Najran University, P.O. Box 1988, Najran 61441, Saudi Arabia; e Departments of Pediatrics, College of Medicine, Najran University, Najran P.O. Box 1988, Saudi Arabia; f Critical Care Nursing, Department of Medical Surgical Nursing, Najran University, Saudi Arabia; g College of Nursing, Prince Sattam bin Abdulaziz University, Al-Kharj 11942, Saudi Arabia; h Department of Nursing Administration, Faculty of Nursing, Port Said University, Egypt; i Department of Medical Biochemistry, Faculty of Medicine, Mansoura University, Mansoura 35516, Egypt; j Department of Basic Medical Sciences, College of Medicine, AlMaarefa University, Diriyah, 13713, Riyadh, Saudi Arabia; k Family and Community Health Nursing Department, Faculty of Nursing, Port Said University, Port Said, Egypt

**Keywords:** Aphrodisiac, Consumption, Married men, Prevalence

## Abstract

**Background:** The prevalence and patterns of aphrodisiac drug consumption without prescription among men in Saudi Arabia remain underexplored, with limited empirical evidence available. Given the potential health implications and societal considerations, a comprehensive investigation is warranted. **Aim:** Assess the Prevalence, pattern of use and the associated factors of Aphrodisiac drugs consumption without prescription among men at Najran City, Saudi Arabia. **Methods:** Employing a cross-sectional descriptive study, 500 participants were included through convenience sampling. The utilized questionnaires covered a range of data, including socio-demographic information, patterns of aphrodisiac use, knowledge about aphrodisiacs, lifestyle details, a sexual health inventory for men, and a perceived stress level scale. **Results:** The study reveals a significant prevalence of unsanctioned aphrodisiac drug use (31%) among men in Najran City, Saudi Arabia, with a majority (79.3%) consuming these substances four times monthly. Associated disparities in knowledge, lifestyle, stress, and sexual function underscore the urgent need for policy interventions and tailored health education initiatives for this demographic. **Conclusion:** Approximately one-third of the sampled population engaged in the unsanctioned use of aphrodisiac drugs, with the majority utilizing them four times monthly. Tablets emerged as the most prevalent form of consumption. Commonly cited motives and justifications included peer influence and the perceived safety of aphrodisiacs. Influential factors encompassed levels of knowledge, lifestyle, stress levels, erectile function, age, education, and the number of wives. **Recommendations:** Urgent policy interventions are warranted to regulate the acquisition and distribution of aphrodisiacs. Tailored health education initiatives should be implemented for married and prospective married men.

## 1 Introduction

Given the paramount importance of sexual health and function in life, the pursuit of remedies to enhance sexual performance has become a prevailing concern. As per estimates derived from the US National Health and Social Life Survey, approximately 31 % of men encounter sexual dysfunction at some point in their lives. Globally, this significant public health issue affects 10–52 % of males and 25–63 % of females. A common avenue for seeking heightened enjoyment in this context is through the usage of aphrodisiacs, often referred to as sex enhancers (Manortey et al., 2018).

A study conducted in Saudi Arabia with 1176 participants, including 1008 sexually active individuals, found that 39.9 % of sexually active participants had used sex-enhancing medications (S-EM), such as herbal or phosphodiesterase type 5 inhibitors (PDE5i) at some point. Notably, 67.2 % of S-EM users were aged ≤ 45 years, indicating a higher prevalence among a younger demographic. When comparing S-EM users and non-users, higher smoking rates were observed among users (55.2 % vs. 25.7 %), and a greater proportion of S-EM users reported having more than one sexual partner (7.5 % vs. 1 %). While both groups mostly had a single sex partner (96.4 %), S-EM users showed a significantly higher prevalence of chronic health problems (35.8 % vs. 27.7 %) (Ahmed et al., 2017). It's important to acknowledge that underreporting may have influenced the accuracy of prevalence data, especially in the context of this study in Arab worlds.

Aphrodisiacs (ADs) refer to chemical substances with the capability to enhance sexual desire, pleasure, or behavior, exhibiting potential therapeutic effects in addressing early-stage sexual dysfunction. These substances can be categorized based on their effects: those amplifying desire, enhancing sexual satisfaction, and maximizing potency. ADs derive from diverse sources, including synthetic materials, foods, plants, and animals. Notably, married men often use aphrodisiacs as pleasure stimulants, deviating from the conventional categories associated with addressing erectile dysfunction (Hafez et al., 2022).

Between 2007 and 2014, the United States Food and Drug Administration (FDA) identified 572 contaminated supplements, with 41.6 % of them found to contain sex-stimulating drugs (Ocloo, 2015). The adulteration of these products poses significant health risks, including kidney failure, vision and hearing issues, heart attacks, cerebrovascular hemorrhage leading to stroke, sudden cardiac death, and persistent erections leading to impotence. Despite the FDA expressing concerns about the safety of unregistered sex-enhancing products, their usage remains robust, persisting despite the documented adverse side effects (Srikanth et al., 2015, Ullah et al., 2023).

The factors associated with aphrodisiac use were summarized by Yidana et al. (2019). These encompass biological causes such as aging and illness, with social considerations playing a significant role. Peer influence, particularly among friends, was identified as a key motivator for aphrodisiac use. The experimentation with aphrodisiacs, especially among youths, was attributed to the application of acquired knowledge. The widespread awareness and consumption of aphrodisiacs were further fueled by advertising through various media channels. Additionally, the heightened usage of aphrodisiacs was linked to psychological factors, including the pursuit of multiple sexual partners, satisfaction, using them as a form of punishment, and eliciting positive responses from women (Yidana et al., 2019).

The consumption of aphrodisiac drugs without a prescription among men poses significant complications, encompassing both health and relational dimensions. Unregulated ingredients in these substances may lead to adverse health effects, ranging from allergic reactions to cardiovascular issues (Amoah et al., 2022). Such self-medication practices may also contribute to a delay in diagnosing underlying health conditions, potentially exacerbating existing medical issues. Psychologically, the unsanctioned use of aphrodisiacs may foster dependency, creating concerns of addiction and affecting overall well-being. Socially, relationships may face strain due to a partner feeling excluded or worried about the health risks involved. Moreover, the legal implications of obtaining these drugs without proper authorization can lead to fines or legal consequences (Baracaldo-Santamaría et al., 2022). To mitigate these risks, it is crucial for individuals to seek professional medical guidance and obtain prescriptions, ensuring both their safety and the efficacy of any treatments. This approach aligns with the importance of responsible healthcare practices in addressing sexual health concerns (Amoah et al., 2022).

### 1.1 Problem statement

In Asia, in contrast to Europe, male sexual dysfunctions (MSDs) often go unnoticed and untreated due to stringent cultural and religious beliefs, socioeconomic factors, and a lack of awareness, potentially leading to an increased reliance on aphrodisiac medications (Irfan et al., 2020). A study conducted in the Kingdom of Saudi Arabia (KSA) highlighted the growing issue of over-the-counter or consumer-directed sales of aphrodisiac drugs (Suleiman et al., 2016). The investigation of the prevalence of sexual stimulant use, its influencing factors, and usage patterns among men in the Middle East remains an area requiring extensive research, as results may vary across societies due to diverse influencing factors such as socioeconomic standards, demographics, race, ethnicity, as well as cultural and religious diversity (Ahmed et al., 2017).

### 1.2 Significance of the study

The significance of studying the consumption of aphrodisiac drugs without a prescription among men in Saudi Arabia cannot be overstated. This research addresses a critical gap in understanding a prevalent but understudied aspect of men's health in the region. By delving into the patterns and prevalence of unsanctioned aphrodisiac use, the study provides valuable data essential for shaping public health policies and interventions. Understanding the motivations and factors associated with such behavior is crucial not only for safeguarding individual health but also for fostering a more comprehensive approach to sexual health in the cultural context of Saudi Arabia. Moreover, the findings contribute to the broader discourse on healthcare accessibility, as the study identifies potential barriers and highlights the need for targeted health education initiatives. Ultimately, this research serves as a foundational step towards promoting informed decision-making, regulatory measures, and culturally sensitive interventions to enhance the overall well-being of men in Saudi Arabia. In this context, our study aims to assess the factors contributing to the increasing use of aphrodisiacs among men in Najran city and its associated determinants.

## 2 Aim of the study

To assess the prevalence, pattern of use and the associated factors of aphrodisiac medication consumption without prescription among married men at Najran City, Saudi Arabia.

### 2.1 Research objectives

1. Assess the prevalence of aphrodisiac drugs use among married men in Najran City, Saudi Arabia, without a prescription
2. Identify the pattern of aphrodisiac medication drugs among married men at Najran City, Saudi Arabia.
3. Explore the associated factors of aphrodisiac drugs consumption without prescription among married men at Najran City, Saudi Arabia.

## 3 Methods

### 3.1 Research design

The study design was a cross-sectional study, chosen for its practicality in capturing a snapshot of the consumption of aphrodisiac drugs without a prescription among men in Saudi Arabia at a specific point in time. This design facilitated the simultaneous collection of data from a diverse group of participants, providing a comprehensive overview of the prevalence, patterns, and associated factors of aphrodisiac drug use in the region. Despite its limitations in establishing causation or tracking changes over time, the cross-sectional approach proved efficient and cost-effective in examining the immediate dynamics of unsanctioned aphrodisiac consumption among the studied population.

### 3.2 Research setting

The study was conducted in Najran, a city located in southwestern Saudi Arabia near the Yemeni border and serving as the capital of Najran Province. This choice of location was driven by Najran's compelling blend of historical significance and rapid modernization, making it a captivating focal point for research. With its population experiencing a significant surge over the years — from 47,500 in 1974 to an impressive 505,652 in 2017 — Najran presents a dynamic urban landscape that encapsulates the essence of societal evolution. The city's geographical proximity to Yemen adds an additional layer of complexity, creating a unique fusion of Saudi Arabian and Yemeni influences that permeates various aspects of daily life. The research endeavors to delve into the multifaceted dimensions of Najran's growth, exploring the factors influencing its population expansion and the intricate interplay between tradition and modernity in the context of urban development.

### 3.3 Study participants

The research study focused on married men, residing in a dynamic urban landscape, to explore the intricate dynamics of married life and the factors influencing individuals within this context. The researchers meticulously determined the sample size, using a carefully calculated formula to strike a balance between obtaining comprehensive insights and navigating logistical constraints associated with studying this particular demographic. The selected sample size for this study amounted to 500 participants, aiming to capture a diverse array of experiences and perspectives within the married male population.

In establishing the inclusion criteria, participants were required to have been married for at least one year, ensuring a sufficient duration for the manifestation of experiences and challenges associated with longer-term marital relationships. Additionally, participants needed to be in full residence with their wives, emphasizing the significance of cohabitation in the context of the study. The exclusion criteria were equally precise, excluding individuals diagnosed with diseases related to the reproductive system, those married for less than a year, and those whose wives did not permanently reside with them for any reason. These criteria were instrumental in refining the target population for the study.

**The determination of the sample size was based on a formula:**

$$n=\frac{z^{2}\times \hat{p}\left(1-\hat{p}\right)}{\varepsilon^{2}}$$

$$n=\frac{1.96^{2}\times 0.5\left(1-0.5\right)}{0.5^{2}}$$

where

*n* represents the required sample size,
*Z* is the Z-score corresponding to the desired level of confidence,
*P* is the estimated population proportion,
*E* is the desired margin of error.

The researchers took deliberate measures to mitigate potential bias in the sampling process, recognizing the importance of obtaining a representative and diverse sample. Despite employing a convenient sample approach, efforts were made to enhance the fairness and reliability of the study's findings. To address potential bias, the researchers ensured that participants were selected from various demographic backgrounds and geographic locations within the designated urban area. This geographical diversity aimed to capture a more comprehensive cross-section of experiences among married men, reducing the risk of skewed or unrepresentative results.

Moreover, the researchers employed a combination of face-to-face interviews and online questionnaire distribution to further minimize potential biases. Conducting face-to-face interviews allowed for personal engagement, enabling the researchers to reach individuals who might be less inclined to participate in an online format. Simultaneously, the option of an online questionnaire using Google Forms catered to participants who preferred or were more accessible through digital means. This dual-method approach aimed to mitigate selection bias by accommodating different communication preferences and lifestyles among the target population.

By implementing these strategies, the researchers sought to foster a more inclusive and unbiased representation of the married male population in the urban setting under study. These considerations in participant selection and engagement were integral to the overall research design, enhancing the credibility and validity of the study's findings.

### 3.4 Tools of data collection

The comprehensive data collection for this research utilized structured questionnaires designed to capture a nuanced understanding of various aspects related to married men in the urban setting under study. The first questionnaire, adapted from Hafez et al. (2022), comprised four distinct parts. The socio-demographic section included items gathering information on age, educational level, economic status, and the number of wives. The second section focused on the pattern of aphrodisiac use, encompassing frequency, personal reasons, and the types of drugs used. The third part involved a knowledge questionnaire covering aspects such as aphrodisiac drug overview, types, indications, contraindications, side effects, and complications. The final part delved into participants' lifestyles through a questionnaire containing 12 points, each scored based on the frequency of adherence. This multi-faceted approach aimed to comprehensively explore the various dimensions influencing married men's health and well-being.

The second questionnaire employed in this research was the Sexual Health Inventory for Men (SHIM) questionnaire, adapted from Rosen et al. (1999). This tool, consisting of five questions with multiple choices, assessed erectile dysfunction. Scores ranging from 1 to 25 were assigned, with higher scores indicative of a lower level of erectile dysfunction. The inclusion of the SHIM questionnaire added a quantitative measure to the investigation, providing valuable insights into a crucial aspect of the participants' sexual health.

The third questionnaire focused on perceived stress levels and was adopted from Cohen and Williamson (1988). Comprising 10 questions with a range of possible choices, this tool aimed to gauge participants' stress perceptions. The scoring system allowed for the categorization of stress levels into low, moderate, and high. This questionnaire contributed a psychological dimension to the research, exploring how stress may intersect with other factors affecting the participants' overall well-being.

The validity assessment of the data collection tools in this study involved a rigorous process carried out by five experts in the fields of public health and community nursing. This panel of experts, possessing specialized knowledge and experience in relevant disciplines, played a crucial role in evaluating the face and content validity of the instruments. Face validity, which assesses the extent to which the tools appear to measure what they intend to measure, was likely established through the expert panel's review. Their collective expertise would have ensured that the tools were visually and intuitively aligned with the concepts being measured, contributing to the overall face validity of the instruments. Content validity, assessing the adequacy and relevance of the items in capturing the targeted constructs, would have been meticulously examined by the expert panel. Their in-depth understanding of public health and community nursing allowed them to critically assess the clarity, appropriateness, and comprehensiveness of the items, ensuring that the tools effectively covered the dimensions relevant to the study.

The involvement of experts in the validation process adds a layer of credibility and rigor to the study. Their input not only enhances the validity of the instruments but also attests to the tools' relevance and appropriateness within the specific context of public health and community nursing. The endorsement from this expert panel strengthens the overall validity of the data collection tools, contributing to the robustness of the study's methodology.

The structured interview's reliability, used for general knowledge and lifestyle assessment, was confirmed with a Cronbach's alpha of 0.83. The SHIM questionnaire demonstrated high reliability with an alpha coefficient of 0.91, as reported by Shamloul et al. (2004). The perceived stress scale, adapted for this research, showed a reliable Cronbach's alpha coefficient of 0.78 according to Anwer et al. (2020). These rigorous assessments of validity and reliability added a robust foundation to the data collection tools, enhancing the credibility of the study's findings.

### 3.5 Pilot study

The feasibility of the study, the responsiveness of subjects, and the time required for data collection were systematically evaluated through a pilot study involving 50 participants. Importantly, the individuals included in the pilot trial were distinct from the main sample participants, safeguarding the independence and integrity of the primary study. This preliminary investigation, initiated with the objective of refining the research design, allowed researchers to scrutinize participant responses to the structured questionnaires. The insights gained from the pilot study were instrumental in identifying and addressing potential challenges, refining the survey instruments for clarity, and ensuring the precision of data collection in the subsequent main study. Additionally, the pilot study served as a strategic tool for estimating the time needed for data collection per participant, offering valuable foresight into logistical considerations. Through this methodical and iterative process, the researchers systematically fine-tuned their approach, bolstering the reliability and robustness of the data collection tools and optimizing the overall efficiency of the subsequent main sample study.

### 3.6 Ethical consideration

Before commencing with the data collection phase, the research adhered to rigorous ethical standards, securing approval from the ethics committee to ensure the welfare and confidentiality of participants. Stringent principles of research ethics were strictly followed throughout the study. In instances of face-to-face interviews, the researchers prioritized transparency by elucidating the study's purpose to participants. Emphasis was placed on assuring individuals that any information disclosed during the interview would remain confidential, exclusively utilized for research purposes. Conversely, in the case of the online technique, these assurances were clearly outlined at the onset of the questionnaire, creating a transparent foundation for participants.

Respecting the autonomy and informed consent of the participants, the researchers underscored the confidentiality of their responses and articulated that the gathered information would be utilized exclusively for research needs. This approach aimed to cultivate an environment of trust, ensuring that participants felt secure in their decision to contribute to the study. Implicit assent from participants was inferred upon their voluntary completion of the questionnaires, signifying their understanding and agreement with the ethical guidelines presented. By upholding these ethical standards and fostering clear communication throughout the research process, the study prioritized the well-being and rights of the participants, establishing a foundation of integrity in the pursuit of valuable insights.

### 3.7 IV – Statistical design

Following the meticulous collection of data, a systematic process of organization, tabulation, and statistical analysis ensued, utilizing IBM's SPSS version 19 (Statistical Package for Social Studies) based in Illinois, Chicago, USA. The collected data were subjected to comprehensive examination through various statistical procedures to derive meaningful insights. The organization involved structuring the data in a coherent manner, facilitating subsequent analyses. Tabulation, a crucial step in this process, allowed for a clear presentation of patterns, trends, and frequencies within the dataset.

Statistical analyses were conducted with precision to unveil patterns and relationships between variables. Distributions by percentage and number were computed to provide a comprehensive understanding of the prevalence and frequency of different responses or outcomes within the data set. The utilization of the T test and X2 allowed for the exploration of statistical differences between variables, shedding light on significant associations or variations.

The chosen statistical significance threshold was set at p 0.05, indicating that any observed differences or relationships between variables were considered statistically significant if the probability of such occurrences happening by chance was less than 5 %. This rigorous criterion ensured a robust and reliable interpretation of the results, instilling confidence in the validity of the study's findings.

By employing SPSS and adhering to established statistical methodologies, the researchers equipped themselves with a powerful tool to unravel the complexities embedded in the collected data. This analytical approach not only facilitated a meticulous exploration of patterns but also enabled the identification of statistically significant relationships, contributing to the overall credibility and rigor of the research.

## 4 Results

Table 1 provides a comprehensive overview of the frequency distribution within the studied sample concerning socio-demographic characteristics, encompassing 500 participants. The age distribution reveals a predominant representation in the 40–49 age group, constituting 66.6 % of the total sample. Participants aged 30–39 and those aged 50 and above make up 10 % and 23.4 % of the sample, respectively. This distribution underscores a significant concentration within the middle age range. Educational background within the sample demonstrates a diverse representation. The majority, comprising 46.8 %, has completed secondary education, followed by those with a bachelor's degree at 22.2 %. Participants with the ability to read and write account for 24 %, while 7 % of the sample is categorized as illiterate. This diversity in educational attainment contributes to a well-rounded demographic representation within the study. Regarding the marital status of the participants, the majority report having a single wife, constituting 62.6 % of the sample. In contrast, 37.4 % of participants are married to more than one wife. This distribution sheds light on the varied marital structures present within the study population, providing a basis for further exploration into the dynamics of married life in the context of the research objectives. Overall, Table 1 serves as a foundational snapshot of the socio-demographic characteristics of the studied sample, laying the groundwork for subsequent analyses and interpretations.

**Table 1:** Frequency distribution of the studied sample regarding socio-demographic data (n = 500).

Items	N	%
**Age**		
30–39	50	10
40–49	333	66.6
≥50	117	23.4
**Education**		
Illiterate	35	7
Read and write	120	24
Secondary education	234	46.8
Bachelor degree	111	22.2
**Number of wives**		
1	313	62.6
>1	187	37.4

**Fig. 1:**
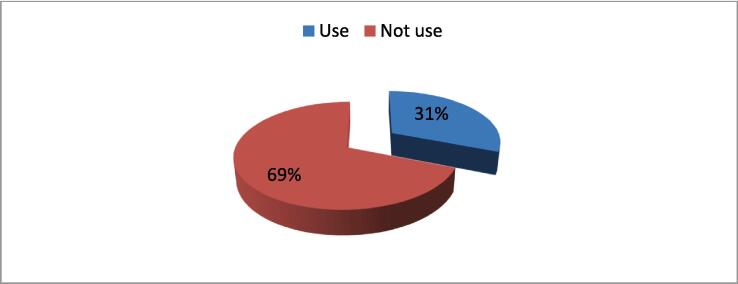
Frequency distribution of the studied sample regarding their use to aphrodisiacs (n = 500).

Fig. 1 illuminates a significant aspect of the study, revealing that 31 % of the studied group reported the usage of aphrodisiacs. This finding underscores the prevalence of aphrodisiac use within the demographic under investigation. The graphical representation provides a clear and concise snapshot, offering an immediate visual insight into the proportion of participants engaging in this particular behavior. This percentage not only highlights the relevance of aphrodisiac consumption within the studied population but also serves as a pivotal point of interest for further exploration into the patterns, motivations, and potential implications associated with the utilization of aphrodisiacs among the participants. As a focal point in the study's results, Fig. 1 sets the stage for a more in-depth analysis and interpretation of factors influencing aphrodisiac usage within the context of the broader research objectives.

Table 2 meticulously details the frequency distribution of the studied sample (n = 155) concerning the pattern of aphrodisiac use. The findings highlight a nuanced landscape of aphrodisiac consumption, revealing that 79.3 % of participants utilize these substances less than four times a month. Motivations for use vary, with 40.7 % expressing a desire for a sense of control and confidence during marital relationships, while 26.7 % imitate a friend's usage. The temporal dimension of aphrodisiac use is diverse, spanning less than a year for 43.3 % of participants, 1–2 years for 40.7 %, and over 3 years for 19.3 %. Friends significantly influence usage recommendations (60.0 %), followed by advertisements (26.7 %) and advice from pharmacists (16.7 %). Factors influencing the choice of aphrodisiacs include safety (54.7 %), advice from friends (28.0 %), and availability (20.7 %). Tablets emerge as the most prevalent form (74.7 %), followed by sprays (20.7 %) and creams (8.0 %). Table 2 thus unveils a complex interplay of factors shaping aphrodisiac consumption patterns, setting the stage for a comprehensive exploration of motivations and preferences within the studied demographic.

**Table 2:** Frequency distribution of studied sample regarding Pattern of aphrodisiac use (n = 155).

Items	N	%
**How many times a month**		
<4 time	119	79.3
4–6 times	15	10
>6 times	21	14
**Personal reasons for use**		
An imitation of a friend	40	26.7
A sense of control and confidence during the marital relationship	61	40.7
To satisfy my sexual needs	29	19.3
**History of use**		
Less than 1 year	65	43.3
1–2	61	40.7
More than 3 years	29	19.3
**Who recommend you to use (multiple responses considered)**		
Friend	90	60.0
Advertisement	40	26.7
Advice from pharmacist	25	16.7
**Factors affecting your choice to the drug (multiple responses is considered)**		
Safe	82	54.7
Advice from friend	42	28.0
Available	31	20.7
**Most common type**		
Tablet	112	74.7
Cream	12	8.0
Spray	31	20.7

Table 3 offers a revealing comparison between participants who use aphrodisiacs and those who abstain from them, focusing on mean scores related to lifestyle, stress level, knowledge, and erectile dysfunction. The data portrays significant distinctions, emphasizing the potential impact of aphrodisiac usage on various aspects of participants' well-being. Notably, those using aphrodisiacs demonstrate a lower knowledge score, suggesting disparities in awareness. Lifestyle differences are evident, with users scoring lower, possibly indicating a connection between aphrodisiac use and specific lifestyle choices. Higher stress levels among aphrodisiac users imply a potential psychological dimension to usage. Intriguingly, users exhibit a lower mean score for erectile dysfunction, suggesting a potential association with reduced prevalence. The consistent statistical significance (T and P values of 0.000) underlines the robustness of these findings, urging further exploration into the complex dynamics between aphrodisiac consumption and overall well-being within the studied population.

**Table 3:** Comparison between the studied sample who use and the studied sample who don’t use aphrodisiacs regarding the mean score of life style, stress level and the erectile dysfunction.

Items	Use	Not use	T	P
Knowledge	10.03 ± 2.4	12.6 ± 1.3	12.1	0.000
Life style	19.7 ± 4.2	28.6 ± 4.3	21.3	0.000
Stress level	26.1 ± 6.6	21.4 ± 7.3	6.8	0.000
Erectile dysfunction	17.9 ± 5.1	21.3 ± 2.5	7.8	0.000

Table 4 presents a detailed examination of the relationship between the use of aphrodisiacs and various demographic factors within the studied sample of 500 participants. The findings highlight significant associations, shedding light on potential influences shaping aphrodisiac consumption patterns. Age emerges as a notable determinant, with participants aged 40–49 exhibiting the highest percentage of aphrodisiac use at 75 %, followed by those aged 50 and above at 50.4 %. This suggests an age-related influence on the likelihood of aphrodisiac utilization. Education level also plays a pivotal role, with higher percentages of use observed among illiterate individuals (85.7 %) and those with basic reading and writing skills (70.8 %). Conversely, participants with secondary education or a bachelor's degree show lower percentages of aphrodisiac use, indicating a potential link between lower educational attainment and a higher propensity for consumption. The marital structure, as indicated by the number of wives, reveals a significant association with aphrodisiac use. Participants with more than one wife demonstrate a considerably higher percentage of aphrodisiac use (89 %) compared to those with only one wife (61 %). This relationship suggests that marital dynamics may influence the prevalence of aphrodisiac utilization within the studied population. The substantial chi-square values and low p-values underscore the statistical significance of these associations. Overall, Table 4 provides valuable insights into the demographic factors influencing aphrodisiac use, paving the way for deeper investigations into the complex interplay between personal characteristics and this specific health-related behavior within the studied demographic.

**Table 4:** The relation between studied sample use and demographic factors (n = 500).

Items	Total	Use		Not use		X^2^	P
		N	%	N	%		
**Age**						32.2	0.000
30–39	50	16	32	34	68		
40–49	333	75	22.5	258	77.5		
≥50	117	59	50.4	58	49.6		
**Education**						215.9	0.000
Illiterate	35	30	85.7	5	14.3		
Read and write	120	85	70.8	35	29.2		
Secondary education	234	15	6.4	218	93.6		
Bachelor degree	111	20		91			
**Number of wives**						44.03	0.000
1	313	61		152			
>1	187	89		98			

## 5 Discussion

The pursuit of enhanced sexual well-being has been a persistent aspect of human history, with individuals seeking various methods to improve, maintain, or restore their sexual prowess and desire. Recognizing the paramount importance of sexual health to overall well-being (Flynn et al., 2016), the quest for treatments to enhance sexual function has been a longstanding preoccupation. Notably, sexual dysfunction is prevalent, affecting 31 % of men at some point in their lives according to data from the US National Health and Social Life Survey (Ramanathan & Redelman, 2020). This issue extends globally, impacting 10–52 % of males and 25–63 % of females, signifying a substantial public health concern.

In response to this pervasive concern, individuals have commonly turned to aphrodisiacs or sexual enhancers (Çayan et al., 2017) as a means of attaining sexual gratification. Against this backdrop, the current study aims to assess the prevalence, patterns of use, and associated factors of aphrodisiac medication consumption without prescription among married men in Najran City, Saudi Arabia. By delving into the prevalence and behaviours surrounding the use of aphrodisiacs, the research seeks to contribute valuable insights into the dynamics of sexual health in this specific demographic, addressing a significant and prevalent aspect of human well-being.

The results of the current study reveal that nearly one third of the studied sample acknowledges the use of aphrodisiacs to enhance sexual desire and satisfaction, with a significant majority using these substances more than four times per month. The researchers suggest that the age distribution within the sample, primarily concentrated between 40 and 49 years old, may be a contributing factor to the prevalence of aphrodisiac use. This age range corresponds to a period where physiological changes associated with aging can lead to a decline in sexual desire and effectiveness. This aligns with the common understanding that as individuals age, there tends to be a decrease in arousal and sexual desire.

Furthermore, the level of education emerges as another potential factor influencing aphrodisiac use, with a notable proportion of the sample having only a secondary education. This suggests a potential association between lower educational attainment and a higher likelihood of using aphrodisiacs without being fully informed about their negative effects.

Additionally, the study suggests that having multiple wives could be a contributing factor to the consumption of aphrodisiacs, as slightly more than one-third of the sample had entered into multiple marriages. Table 4 further validates these potential consequences associated with the utilization of aphrodisiacs, affirming the complex interplay between demographic factors and aphrodisiac consumption patterns within the studied population. These findings underscore the need for targeted interventions and education to promote informed and responsible usage of aphrodisiacs, especially among demographics more prone to their use.

The findings of the current study align with and find support in the broader scholarly literature on aphrodisiac usage. Hafez et al. (2022) substantiated these results, highlighting that slightly fewer than one-third of individuals in their study utilized aphrodisiacs. Similarly, Wu et al. (2017) conducted a study involving 3485 male consumers, revealing that a third of the sample engaged in aphrodisiac use. Amoah et al. (2022) contributed to this body of knowledge by reporting that a little over a quarter of their participants acknowledged using aphrodisiacs in the six months preceding their research.

In contrast, Adamu et al. (2022) conducted a study in the Sokoto metropolis, finding that aphrodisiac usage is quite common among adults, with slightly more than two-thirds of the participants using them, and up to two-fifths having done so in the previous month. Manortey et al. (2018) also reported that somewhat more than half of their sample group utilized aphrodisiacs. Furthermore, Wada et al. (2023) uncovered that about two-thirds of adults in northern Nigeria engage in the practice of using sexual enhancement, emphasizing the widespread nature of this phenomenon.

The consistency of these findings across diverse studies and populations underscores the prevalence and significance of aphrodisiac usage in various cultural and geographical contexts. These collective insights contribute to a comprehensive understanding of the patterns and prevalence of aphrodisiac consumption, providing a valuable foundation for further research and interventions in the realm of sexual health.

The current study's findings revealed that roughly two fifths of the sample used aphrodisiacs to feel in control and confident during marriage, slightly more than one quarter did so to imitate a friend, and roughly one fifth did so to satiate their own sexual needs. According to the researcher, one of the factors contributing to the spread of such drugs may be the lack of knowledge due to low educational attainment, as the vast majority of the study group has low or medium levels of education, as well as the lack of awareness of the immediate danger of continued, excessive use of these drugs. This could be a result of being removed from Islamic teachings, having a lot of temptations in life, and having negative friends, which could also play a part in why people use these sexual enhancers. The spread of such drugs may also be attributed to advanced age and polygamy.

The association between aphrodisiac usage and various factors has been explored by Yidana et al. (2019), who identified a significant relationship between the use of aphrodisiacs and elements such as one's faith, marital status, age, diseases, male organ size, peer pressure, maintaining multiple sexual partners, and satisfying women sexually. This multifaceted connection underscores the complexity of factors influencing aphrodisiac consumption, ranging from personal beliefs and health considerations to societal and interpersonal dynamics.

Wu et al. (2017) further delved into the independent links between aphrodisiac use and demographic and health-related factors. Their study revealed that aphrodisiac use was independently associated with older age, ethnic minorities, low education, HIV infection, chronic illness, and inconsistent condom use in commercial sex. The findings suggest that older male clients, in particular, perceived aphrodisiacs as enhancing their sexual performance, leading to risky behaviors in commercial sex contexts. This connection underscores the potential implications of aphrodisiac use in the context of high-risk behaviors for HIV transmission and infection within certain communities.

The collective insights from these studies highlight the intricate interplay of individual, cultural, and health-related factors that contribute to aphrodisiac consumption. Understanding these relationships is crucial for developing targeted interventions and health promotion strategies that consider the diverse influences shaping attitudes and behaviors related to sexual health and the use of aphrodisiacs.

Building on the intricate web of factors influencing aphrodisiac usage, Manortey et al. (2018) contribute valuable insights by highlighting that usage tends to be higher among individuals with lower educational levels. This correlation underscores the importance of educational attainment as a determinant in aphrodisiac consumption patterns, suggesting that individuals with lower levels of formal education may be more prone to engaging in the use of these substances.

Moreover, the study conducted by Manortey et al. (2018) brings attention to several other factors that interplay with aphrodisiac usage. Notably, statistics revealed a correlation between aphrodisiac use and the number of sexual partners, the prevalence of sexual issues, exposure to advertising, and awareness of negative effects. These findings elucidate the multifaceted nature of the influences shaping the decision to use aphrodisiacs, ranging from interpersonal dynamics and sexual health issues to external stimuli such as advertising.

Similarly, Amoah et al. (2022) provide a broader socio-ecological perspective by identifying community-level, interpersonal, public policy, and organizational/institutional factors influencing the sale and use of aphrodisiacs among men in Ghana. Social norms, expectations within communities, interpersonal dynamics with friends and partners, drug-related regulations, and health system arrangements collectively contribute to the landscape of aphrodisiac consumption.

Together, these studies emphasize the need for comprehensive interventions that address the diverse factors influencing aphrodisiac usage, recognizing the complex interplay between individual characteristics, education, interpersonal relationships, and broader socio-cultural and policy contexts. Such insights are crucial for developing targeted public health strategies to promote informed and responsible aphrodisiac use within diverse populations.

The findings of the present study illuminate the significant influence of friends on aphrodisiac usage, with slightly less than two-thirds of the studied group reporting that they were influenced by their peers to use these substances. This outcome resonates with the researcher's perspective that friends wield a considerable impact on the trajectories and choices of their peers, either positively supporting or potentially causing harm. The study aligns with DeLuca Bishop (2021) observations, which emphasize the influential role of friends and peers in shaping emerging adults' sexual attitudes and behaviors.

Contrastingly, Nikken and de Graaf (2013) reported a different perspective, suggesting that friends' encouragement to utilize sexual media had little bearing on adolescents' subsequent sexual conduct or attitudes. This disparity underscores the nuanced nature of peer influence and its differential effects on various aspects of individuals' behaviors.

Furthermore, Adamu et al. (2022) contributes to this discourse by noting that the majority of aphrodisiac users in their study claimed to have learned about these substances from their acquaintances. This reinforces the idea that social networks, particularly friendships and acquaintances, serve as crucial channels for disseminating information and influencing behaviors related to aphrodisiac consumption.

The study's sample underscores tablets as the most widely used aphrodisiac type, with cream and spray being less commonly utilized. This finding aligns with the results of other research studies, including those conducted by Adamu et al., 2022, Mohammed-Durosinlorun and Mata, 2008, as well as Yidana et al. (2019). These studies collectively reveal that tablets or capsules consistently rank among the most popular aphrodisiac types used by respondents across different populations.

Additionally, the research findings emphasize the significance of native plants alongside tablets/capsules as prevalent choices for aphrodisiac consumption, as reported by Yidana et al. (2019). This variation in preferred aphrodisiac types underscores the influence of cultural and regional factors in shaping the choices made by individuals seeking these substances. Furthermore, the study's sample highlights a predominant mode of administration, with up to 93.5 % of respondents indicating the oral route when asked about the administration of aphrodisiacs. This aligns with a common practice observed across various studies and suggests a cultural tendency toward oral consumption of aphrodisiacs.

The results of the study highlight significant disparities between the overall level of knowledge within the studied group and their use of aphrodisiacs. Furthermore, the data reveal substantial variations between the sample's overall lifestyle and their engagement in the use of aphrodisiacs. Mensah (2018), emphasizing that possessing a good level of knowledge and leading a healthy lifestyle act as protective factors against the use of aphrodisiacs, supports and validates the findings of the present study. This alignment underscores the interconnectedness of knowledge and lifestyle choices in influencing individuals' decisions regarding aphrodisiac consumption.

This correlation between knowledge and behavior is further reinforced by similar findings from other studies. For instance, research by Manortey et al. (2018) has shown that respondents who claimed some level of information about the potential negative effects of aphrodisiac medications were 0.8 times less likely to use the substance compared to their counterparts who admitted having no knowledge. This consistent pattern across studies emphasizes the pivotal role of awareness and understanding of the potential consequences in shaping individuals' choices related to aphrodisiac use.

The findings of the study illuminate a notable connection between the sample's use of aphrodisiacs and the prevalence of erectile dysfunction among them. This correlation underscores the complex interplay between sexual health issues and the decision to engage in aphrodisiac consumption. Additionally, the results reveal strong correlations between the studied sample's perceived level of stress and their use of aphrodisiacs, suggesting a potential link between stress and the resort to these substances.

In parallel, Mensah (2018) study echoes a similar sentiment, noting that the usage of aphrodisiacs is influenced by both the prevalence of sexual issues and exposure to stressful situations. This alignment with Mensah's findings further substantiates the notion that individuals may turn to aphrodisiacs as a coping mechanism or solution to address sexual challenges and navigate stressful circumstances.

Conversely, Atuobi-Bediako’s (2019) study provides an interesting contrast by revealing that 61 % of aphrodisiac users reported having no sexual issues. This suggests that a significant portion of users may engage with aphrodisiacs for leisure or recreational purposes rather than as a response to specific sexual challenges or stressors. This diverse range of motivations highlights the multifaceted nature of aphrodisiac consumption, encompassing both therapeutic and recreational dimensions.

## 6 Conclusion

In conclusion, the findings of this study shed light on the prevalence and patterns of aphrodisiac drug use among the studied sample. Approximately one third of the participants acknowledged using aphrodisiac drugs without a prescription, with the majority using them about four times per month. Tablets emerged as the most commonly used type, and the primary motivations for use were often attributed to influence from friends and perceived safety of these substances.

The study revealed that several factors significantly influenced aphrodisiac use within the sample. Notably, the level of knowledge, lifestyle choices, stress levels, erectile function, age, education, and the number of wives were identified as key influencers. These findings underscore the complexity of factors contributing to aphrodisiac consumption, encompassing both social influences and individual characteristics. The prevalence of aphrodisiac use for recreational purposes, as indicated by the frequency of use and motives cited, suggests a cultural and societal acceptance of these substances. The dominance of tablets as the preferred type further reflects prevailing preferences within the studied population.

Furthermore, the study underscores the need for targeted interventions that consider the influential role of friends, awareness campaigns to enhance knowledge about potential risks, and initiatives promoting healthier lifestyles. Addressing the identified influencers can contribute to informed decision-making and potentially mitigate the unregulated use of aphrodisiac drugs. In essence, this study provides valuable insights into the nuanced landscape of aphrodisiac use, emphasizing the importance of a holistic understanding of individual, social, and cultural factors. Future research and public health initiatives can build upon these findings to develop comprehensive strategies for promoting sexual health and responsible aphrodisiac use within diverse populations.

### 6.1 Recommendations

Based on the study's findings, several recommendations can be put forth to address the implications of aphrodisiac use among the studied population. Firstly, there is a clear need for policy interventions to regulate the purchase and distribution of aphrodisiacs. Implementing regulations and guidelines can help ensure that these substances are obtained through legal and monitored channels, promoting safer usage and minimizing potential health risks associated with unregulated consumption.Also, issuing a law criminalizing the sale and circulation of steroids without a prescription, as is the case with antibiotic. Such policies could include requirements for prescription-only access or stringent quality control measures to safeguard the public.

In conjunction with policy interventions, targeted health education programs should be developed and implemented, specifically focusing on married men and those undergoing premarital preparation. These programs can provide comprehensive information about the potential risks and benefits of aphrodisiac use, emphasizing the importance of seeking professional advice before engaging in self-medication. By enhancing awareness and knowledge, these initiatives aim to empower individuals to make informed decisions regarding their sexual health, fostering a sense of responsibility and minimizing the reliance on aphrodisiacs without proper guidance.

Furthermore, it is crucial to advocate for further research in this domain, encouraging studies with larger sample sizes conducted in diverse settings. A broader and more comprehensive understanding of the factors influencing aphrodisiac use, along with its associated health outcomes, can contribute to the development of evidence-based interventions and policies. Research endeavors in different sociocultural contexts will enrich the existing knowledge base, enabling more tailored approaches to address the complexities surrounding aphrodisiac consumption. Overall, a multifaceted strategy incorporating policy measures, education initiatives, and ongoing research efforts can work synergistically to promote responsible and informed aphrodisiac use within communities.

### 6.2 Study implications

The consumption of aphrodisiac drugs without a prescription among men in Saudi Arabia presents significant health and societal concerns. From a health perspective, individuals engaging in such practices may expose themselves to unknown dosages and potentially harmful ingredients, increasing the risk of adverse effects and complications. Without proper medical supervision, these self-prescribed medications may interact negatively with existing health conditions or other medications, posing a serious threat to individuals' well-being. The study's findings may underscore the importance of reinforcing regulatory measures to ensure the safe distribution of aphrodisiac drugs, advocating for the need for prescription-based access to these medications to safeguard public health.

Beyond health implications, the study sheds light on broader societal factors influencing the consumption of aphrodisiac drugs without medical oversight. Cultural stigma and taboos surrounding sexual health may contribute to individuals seeking self-medication instead of seeking professional advice. Addressing these cultural nuances becomes crucial in developing targeted interventions, including educational programs that destigmatize sexual health issues and encourage open discussions. Moreover, the study may prompt policymakers to consider initiatives aimed at improving overall access to healthcare services, breaking down barriers that prevent individuals from seeking proper medical guidance for sexual health concerns. Ultimately, a holistic approach that combines regulatory measures, cultural awareness, and improved healthcare access may be essential in mitigating the risks associated with the unregulated consumption of aphrodisiac drugs in Saudi Arabia.

## Availability of data and materials

All data are publicly available for sharing and publication. The manuscript does not have any other associated data, and all necessary data have been declared within the original manuscript.

## Declaration of competing interest

The authors declare that they have no known competing financial interests or personal relationships that could have appeared to influence the work reported in this paper.
